# Proteinaceous Molecules Mediating *Bifidobacterium*-Host Interactions

**DOI:** 10.3389/fmicb.2016.01193

**Published:** 2016-08-03

**Authors:** Lorena Ruiz, Susana Delgado, Patricia Ruas-Madiedo, Abelardo Margolles, Borja Sánchez

**Affiliations:** ^1^Department of Nutrition, Food Science and Food Technology, Universidad Complutense de MadridSpain; ^2^Department of Microbiology and Biochemistry of Dairy Products, Instituto de Productos Lácteos de Asturias-Consejo Superior de Investigaciones CientíficasVillaviciosa, Spain

**Keywords:** *Bifidobacterium*, host interaction, proteome, immunomodulation, adhesin

## Abstract

Bifidobacteria are commensal microoganisms found in the gastrointestinal tract. Several strains have been attributed beneficial traits at local and systemic levels, through pathogen exclusion or immune modulation, among other benefits. This has promoted a growing industrial and scientific interest in bifidobacteria as probiotic supplements. However, the molecular mechanisms mediating this cross-talk with the human host remain unknown. High-throughput technologies, from functional genomics to transcriptomics, proteomics, and interactomics coupled to the development of both *in vitro* and *in vivo* models to study the dynamics of the intestinal microbiota and their effects on host cells, have eased the identification of key molecules in these interactions. Numerous secreted or surface-associated proteins or peptides have been identified as potential mediators of bifidobacteria-host interactions and molecular cross-talk, directly participating in sensing environmental factors, promoting intestinal colonization, or mediating a dialogue with mucosa-associated immune cells. On the other hand, bifidobacteria induce the production of proteins in the intestine, by epithelial or immune cells, and other gut bacteria, which are key elements in orchestrating interactions among bifidobacteria, gut microbiota, and host cells. This review aims to give a comprehensive overview on proteinaceous molecules described and characterized to date, as mediators of the dynamic interplay between bifidobacteria and the human host, providing a framework to identify knowledge gaps and future research needs.

## Introduction

The human gut is inhabited by a trillion of microorganisms which constitute the gut microbiota. These microorganisms are in close contact with the intestinal mucosa, which represents the largest extension of the human body exposed to external stimuli. A complex molecular interplay is established among microbiota, dietary components and host cells, which regulates immune and metabolic functions in the host (Furusawa et al., 2015). Dysbiosis, defined as changes in the gut microbiota structure associated to healthy individuals, disrupts the microbiome-host cross-talk homeostasis and correlates with metabolic and inflammatory disorders (Evans et al., 2013; Patel et al., 2013; Levy et al., 2015).

Pro- and pre-biotics can improve host health through microbiota modulation and immune system boosting (Picard et al., 2005). Some strains of bifidobacteria, which are among the first colonizers of the human intestine and one of the dominant groups in the breast-fed infant microbiota (Garrido et al., 2012), have been attributed several health benefits, encouraging interest in their use as probiotics. Pathogen inhibition and diarrhea amelioration are their best established outcomes and have been related to the production of organic acids (Fukuda et al., 2011), antibacterial peptides (Moroni et al., 2006), quorum-sensing inhibitors (Cotar et al., 2010), pathogen displacement (Ruas-Madiedo et al., 2006), and virulence attenuation (Tanner et al., 2016). Bifidobacteria also regulate host functions and ferment complex polysaccharides from our diet (Ménard et al., 2005; Heuvelin et al., 2009, 2010; Bermudez-Brito et al., 2013; Furusawa et al., 2015), although there is still limited knowledge on the molecular mechanisms triggering these effects.

Delineating the specific molecules mediating *Bifidobacterium* cross-talk with the host, will help to understand their beneficial effects and establish microbiome-targeted therapies for human diseases. This review gives an overview on molecules behind the bifidobacterial-host cross-talk, providing a framework to design safe and efficacious probiotic-derived supplements (Licciardi et al., 2010; Shenderov, 2013).

## Intestinal mucosa adhesion

**Figure 1:**
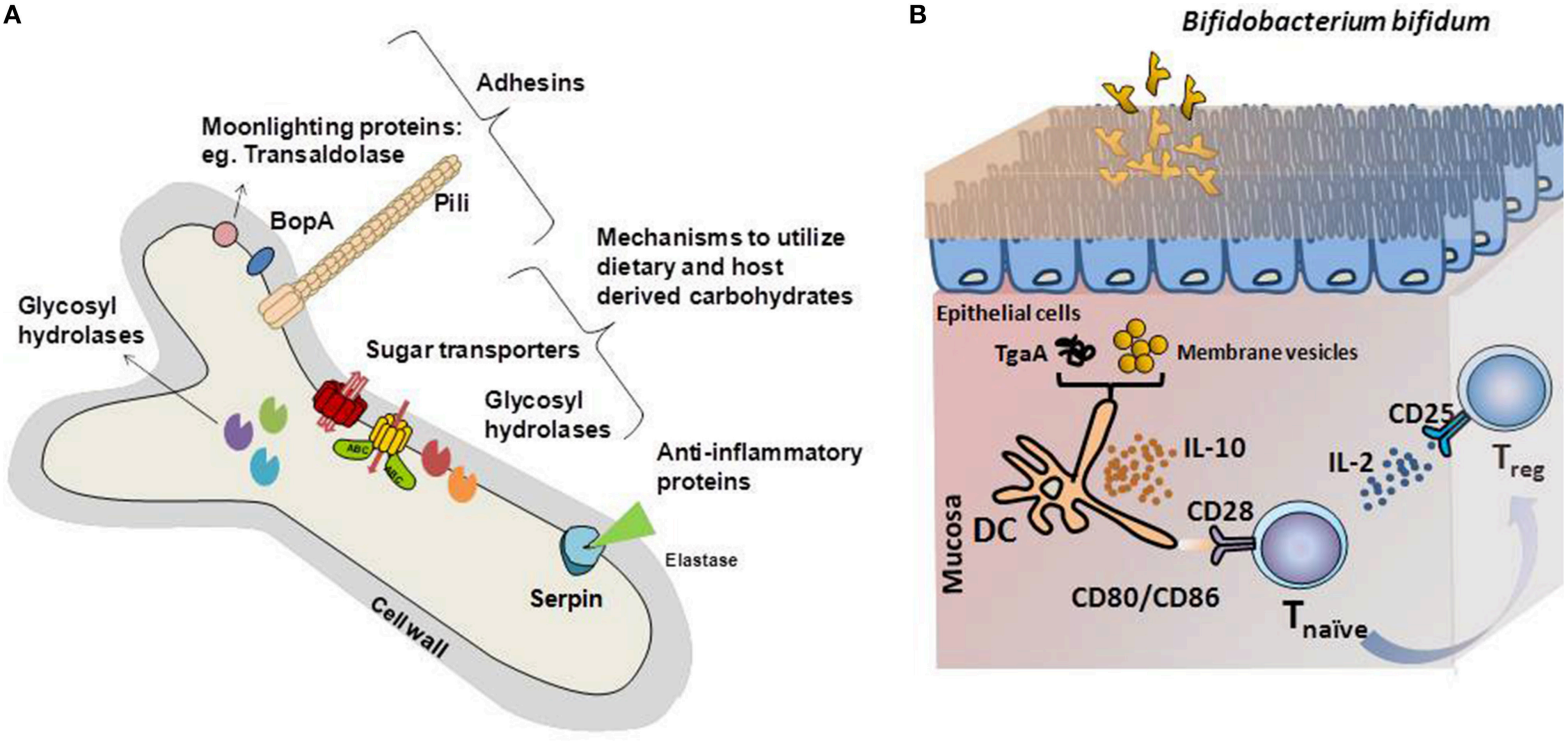
**(A)** Schematic representation of the bifidobacterial proteins identified as key mediators of the cross-talk mechanisms with the intestinal environment. Adhesin-like factors, proteins with immunomodulatory capabilities and glycosyl hydrolases specific for carbon sources encountered in the gastrointestinal tract are represented. **(B)** Graphical illustration of the immunomodulatory mechanisms driven by model *Bifidobacterium bifidum* strains. Different *B. bifidum* fractions and molecules induces T_reg_ response, key in maintaining the balance of effector T-cell responses. Membrane vesicles or the extracellular protein TgaA affects dendritic cells, which induces T_reg_ differentiation after interaction with naïve T-cells. In this process, increased IL-10 secretion, recognition of CD80, and CD86 by CD28 in naïve T-cells and release of IL-2 are key for T_reg_ response development.

Bacterial adhesion to the intestinal surface is mediated by non-specific, hydrophobic or electrostatic interactions, and specific mechanisms involving macromolecular interactions between bacterial and host receptors. Functionally characterized *Bifidobacterium* adhesins, surface-associated proteins that facilitate bifidobacteria attachment to intestinal cells and/or the extracellular matrixes surrounding them, are reviewed below and summarized in Figure 1.

Involvement of pili in bifidobacterial adhesion has been studied in *Bifidobacterium bifidum* and *Bifidobacterium breve* (O'Connell Motherway et al., 2011b; Turroni et al., 2013). The *B. bifidum* PRL2010 genome harbors three pilus clusters. Of these, *pil2* and *pil3*, encode putative sortase-dependent pili that are expressed under both *in vitro* and *in vivo* conditions. Heterologous expression of *pil3* in *Lactococcus lactis* significantly increased this bacterium adhesion to the human epithelial cell line Caco-2 (Turroni et al., 2013). The type IVb tight adherence (Tad) pilus-encoding gene cluster from *B. breve* UCC2003, was found to be essential for the colonization of, and persistence in, the murine gut. Tad inactivation impaired the strain ability to stably colonize the murine intestine, as reflected by reduced shedding level and bifidobacterial numbers in the gut (O'Connell Motherway et al., 2011b). Another surface-anchored protein potentially involved in intestinal adhesion of bifidobacteria is BopA. This purified lipoprotein competes with *B. bifidum* MIMBb75 adhesion to Caco-2 cells, and probably facilitates *B. bifidum* adhesion (Guglielmetti et al., 2008). Indeed, *Bifidobacterium* strains overexpressing *bopA* adhere better (Gleinser et al., 2012). However, *B. bifidum* treatment with anti-BopA antibodies does not reduce the attachment to intestinal cells (Kainulainen et al., 2013), thus the specific adhesion mechanism mediated through BopA must be further elucidated.

Remarkably, bifidobacteria can use some moonlighting proteins, those with multiple functions (Jeffery, 2003), as adhesin-like factors. Surface-exposed glycolytic enzymes, including transaldolase from *B. bifidum* and enolase from *Bifidobacterium animalis*, are adhere to mucin and plasminogen, respectively (Candela et al., 2009; González-Rodríguez et al., 2012). Other surface-exposed moonlighting proteins, including the chaperone DnaK from *B. animalis* and the elongation factor Tu from *Bifidobacterium longum*, showed high affinity for human plasminogen *in vitro* and have been proposed as mediators of intestinal attachment (Candela et al., 2010; Wei et al., 2014).

It is worth highlighting that most of the adhesins herein described have been identified on *in vitro* assays and their relevance for intestinal colonization has not been tested *in vivo*. Since laboratory models do not accurately mimic all the factors that can affect bacterial attachment to the intestinal mucosa (e.g., due to the absence of a mucus layer and resident microbiota), functional confirmation of the adhesion capacity *in vivo* is still required (Ouwehand and Salminen, 2003).

## Tight-junctions

A single layer of epithelial cells provides a selective barrier separating intestinal lumen from subjacent tissues. Tight-junctions (TJ) are multi-protein complexes that control molecule translocation across this barrier (Lee, 2015), and their disruption leads to uncontrolled trafficking of noxious molecules triggering inflammation (Bergmann et al., 2013).

Specific *Bifidobacterium* strains promote TJ enhancing epithelial barrier integrity (Ohland and Macnaughton, 2010; Mokkala et al., 2016). In animal models, *B. bifidum* and *B. longum* strains preserved TJ localization, attenuating intestinal permeability, and decreasing necrotizing enterocolitis incidence (Khailova et al., 2009; Bergmann et al., 2013; Srutkova et al., 2015). Preliminary work suggested that *B. bifidum* metabolites, like acetate, induced TJ expression in intestinal cells (Hsieh et al., 2015). Soluble factors present in *B. longum* lysates or secreted by *B. infantis* or *B. breve* strains, also mediate epithelial barrier maintenance (Ménard et al., 2005; Ewaschuk et al., 2008; Sultana et al., 2013). Further research to identify the specific molecules mediating this TJ promotion is needed.

## Mucus, HMO, and non-digestible carbohydrates degradation

The spatial distribution of bacteria throughout the gastrointestinal tract, is partly controlled by nutrients availability for resident microbiota (Donaldson et al., 2016). Indeed, our wellbeing relates with the nutrient harvesting capability of our gut microbes. These microorganisms, overall, can utilize dietary and host carbohydrates, and glycans produced by other gut bacteria. Indeed, 89 “carbohydrate active enzymes” (CAZyme) have recently been identified in 85% of the microbiomes obtained from 488 individuals (Bhattacharya et al., 2015), suggesting that gut bacteria are highly specialized in using available glycans as their main sustenance. Bifidobacterial genomes are abundant in saccharolytic features whose expression is tightly regulated by available carbohydrates (Khoroshkin et al., 2016), supporting that host glycans were a potent evolutionary force driving their successful gut colonization (Sánchez et al., 2013; Milani et al., 2016).

Numerous studies demonstrated bifidobacteria's capability to use dietary non-digestible oligosaccharides, which is on the basis of the prebiotic concept (Rastall and Gibson, 2015). Glycosyl hydrolases (GH, or glycosidades), many of which are extracellular, have high specificity for the oligosaccharides constituents and cleave the glycosidic bonds (Table 1). Special attention has been paid to the GH acting on human milk oligosaccharides (HMO) since these serve as substrates for bifidobacteria, which are the initial gut colonizers in breast-fed infants. HMO are structurally diverse and composed of several monosaccharides (glucose, galactose, N-acetylglucosamine, fucose, or sialic acid). They mainly consist of a lactose core linked to units (*n* = 0–15) of lacto-N-biose (type I) or to N-acetyl-lactosamine (type II; Smilowitz et al., 2014). Bifidobacteria secrete GH that cleave specific linkages within the HMO molecules and the best characterized are those synthesized by *B. bifidum* which, together with *B. longum* subsp. *infantis*, are two abundant species in breast-fed neonates (Table 1). These species employ different strategies for HMO utilization. Whereas *B. bifidum* has an array of membrane-associated GH, *B. longum* subsp. *infantis* is specialized in the import and intracellular breakdown of HMO (Garrido et al., 2013; Jae-Han et al., 2013). Moreover *B. longum* strains have similar HMO-utilization patterns, whilst *B. bifidum* strains are more diverse with some unable to use fucosylated or sialylated HMO (Garrido et al., 2015). Similarly, the *B. breve* HMO utilization profile is strain dependent and, contrary to *B. bifidum*, some strains consume fucosylated or sialilated HMOs. *B. breve*'s capability to use these HMOs explains its abundance in breast-fed babies (Ruiz-Moyano et al., 2013).

**Table 1:** **Gycosyl hydrolases and sugar transporters characterized and/or described in ***Bifidobacterium*** genus**.

**Glycosyl hydrolases**			
**Substrate^a^**	**Glycosyl hydrolase (family)**	**Bifidobacteria species**	**References**
**NON-DIGESTIBLE DIETARY CARBOHYDRATES**			
α-glycans: palatinose (1 → 6); turanose (1 → 3); maltotriose and maltose (1 → 4) linkages, etc.	α-1,6-glucosidase (GH13)	*B. breve* UCC2003	Pokusaeva et al., 2009; Kelly et al., 2016
Starch and starch-like carbohydrates (pullulan, maltodextrin, etc.)	α-amylases, amylopullanases, etc.	*B. adolescentis* 22L	Duranti et al., 2014
Starch hydrolysates (maltodextrins, malto-OS, isomalto-OS, maltose, etc.)	α-glucosidases, α-amylases, etc.	*B. longum* subsp. *longum* BBMN68	Liu et al., 2015
Plant ginsenoside and cellobiose	β-glucosidase (GH1, GH3)	*B. animalis* subsp*. lactis* AD011	Kim et al., 2012
Isoflavone glycosides (daidzin)	β-glucosidases (GH3)	*B. pseudocatenulatum* IPLA36007	Alegría et al., 2014
β-glucosides (mycotoxins from cereal-based foods)	β-glucosidases	*B. adolescentis* DSM20083	Michlmayr et al., 2015
β-galactans, β-galacto-OS: (1 → 4) linkages	Endogalactanase (GH53)	*B. longum* NCC2705	Hinz et al., 2005
β-galactans (potato)	β-1,4-endogalactanase	*B. breve* UCC2003	O'Connell Motherway et al., 2011a
β-1,3-galactooligossacharides and arabinogalactan	exo-β-1,3-galactanase	*B. longum* JCM1217	Fujita et al., 2014
Arabinoxylan [β-(1,4)-linked xylosyl backbone with arabinosyl side chains]	Arabinofuranohydrolase	*B. adolescentis* DSM20083	van den Broek et al., 2005
β-L-arabinofuranosides	β-L-arabinobiosidase (GH121)	*B. longum* JCM 1217	Fujita et al., 2011
α-1,5-linked arabino-OS	α-L-arabinofuranosidase (GH1)	*B. adolescentis* ATCC 15703	Suzuki et al., 2013
Plant ginsenoside	β-D-xylosidase	*B. breve* K-110	Hyun et al., 2012
Xylo-OS	β-D-xylosidase (GH43)	*B. animalis* subsp*. lactis* BB-12	Viborg et al., 2013
β-(2,1) in short-chain inulin-type fructans, Raffinose	β -fructofuranosidase (GH32)	*B. longum* KN29.1	Bujacz et al., 2011
Flavonoid rhamnoglycosides: (1 → 6) linkage	α-L-rhamnosidase	*B. dentium*	Bang et al., 2015
β-Mannans (plants)	Mannanase (GH5_8)	*B. animalis* subsp*. lactis* Bl-04	Morrill et al., 2015
**HUMAN CARBOHYDRATES: MUCIN AND HMO**			
α-L-Fucosyl termini residues from glycoconjugates	1,2-α-L-fucosidase (GH95)	*B. bifidum* JCM1254	Katayama et al., 2004
Mucin-OS (Core 1 type O-glycans)	Endo-α-N-acetylgalactosaminidase (GH101)	*B. longum* JCM 1217	Fujita et al., 2005
Mucin 2 (Core 3 type O-glycans)	α-N-acetylgalactosaminidase (GH129)	*B. bifidum* JCM1254	Kiyohara et al., 2012
Gastroduodenal mucin (terminal GlcNAcα1-4Gal)	α-N-acetylglucosaminidase (GH89)	*B. bifidum* JCM 1254	Shimada et al., 2015
HMO and lacto-N-tetraose (type I chain)	Lacto-N-biosidase (GH20)	*B. bifidum* JCM1254	Wada et al., 2008
HMO α1,3/4-fucosylated OS	1,3–1,4-α-L-fucosidase	*B. bifidum* JCM1254	Ashida et al., 2009; Ito et al., 2013
HMO and lacto-N-neotetraose (type II chain)	β-galactosidase + β-N-acetylhexosaminidases	*B. bifidum* JCM1254	Miwa et al., 2010
HMO sialylOS	Exo-α-sialidase (GH33)	*B. bifidum* JCM1254	Kiyohara et al., 2011
Fucosylated HMO	α- L-fucosidases (GH29, GH95)	*B. longum* subsp*. infantis* ATCC15697	Sela et al., 2011
HMO (type I chain) + (type II chain)	β-1,3-galactosidase + β-galactosidase	*B. longum* subsp*. infantis* ATCC15697	Yoshida et al., 2012
**SUGAR TRANSPORTERS**			
Arabinoxylo-OS	ABC transporter	*Bifidobacterium animalis* subsp. *lactis* Bl-04	Ejby et al., 2013
Xylo-OSs	ABC transporter	*B. animalis* subsp. *lactis* BB-12	Gilad et al., 2010
Galacto-OS	ABC transporter	*B. breve*	O'Connell Motherway et al., 2011a
β-glucans	ABC transporter	*B. longum* subsp. *infantis*	Zhao and Cheung, 2013
Galacto-OS, HMO, fructo-OS	ABC transporter	*B. longum* subsp*. infantis* ATCC15697	Kim et al., 2012
HMOs, inulin, Galacto-OS	ABC transporter	*B. longum* subsp*. infantis* ATCC15697	Garrido et al., 2011
galacto-N-biose/lacto-N-biose	ABC transporter	*B. longum* JCM1217	Wada et al., 2007
4′-galactosyllactose	ABC transporter	*B. breve* Yakult	Shigehisa et al., 2015
Cellobiose, galacto-OS, isomaltose, maltotriose, melibiose, panose, raffinose, stachyose, xylobiose β-xylo-OS	ABC transporter(s)	*B. lactis* Bl-04	Andersen et al., 2013
lacto-N-biose, galacto-N-biose	ABC-transporter	*B. longum* JCM1217	Suzuki et al., 2008
Fructose	ABC-transporter	*B. longum* NCC2705	Liu et al., 2011; Wei et al., 2012
Ribose	ABC transporter	*B. breve* UCC2003	Pokusaeva et al., 2010
Glucose	Secondary transporter	*B. animalis* DSMZ10140	Briczinski et al., 2008
Fructose	PTS	*B. breve* UCC2003	Mazé et al., 2007
Glucose	PTS	*B. longum* NCC2705	Parche et al., 2007
Glucose	PTS	*B. longum* NCC2705	Parche et al., 2006
Glucose	PTS	*B. animalis* subsp*. lactis*	Briczinski et al., 2008

a *OS, oligosaccharide(s)*.

*Human carbohydrates: mucin and HMO*.

*PTS, phosphotransferase system*.

Some bifidobacteria can also utilize mucins from the mucus layer coating the intestine. Mucin composition and structure resemble that of HMO; consisting of a core of different O-glycans, built on α- and β-linked N-acetyl-galactosamine, galactose, and N-acetyl-glucosamine residues, which can incorporate fucose and sialic acid residues (Tailford et al., 2015). *B. longum* and *B. breve* strains' capability to effectively use mucin carbohydrates, has been confirmed *in vitro* (Ruas-Madiedo et al., 2008). However, GH able to degrade mucins have only been described in *B. bifidum* (Table 1). Indeed, in a comparative genomic study 60% of the GH-encoding genes from *B. bifidum* were predicted to breakdown mucin-like glycans and most of them were exclusively present in this bifidobacterial species (Turroni et al., 2014). Remarkably, other species could use the mono- and oligosaccharides released by *B. bifidum* GH thus evidencing the existence of cross-feeding mechanisms, as it has been demonstrated in *B. breve* and *B. bifidum* co-cultures (Egan et al., 2014).

*Bifidobacterium* capacity to metabolize specific dietary and host-derived carbohydrates is also dependent on the presence of specific sugar transport systems. These are crucial for their competitive establishment in the gut, thus representing one of the molecular mechanisms by which bifidobacteria interact with the host. Import sugar mechanisms in bifidobacteria are herein described (Bottacini et al., 2014).

First, ATP-binding cassette (ABC) systems are active transporters which couple ATP hydrolysis to translocation uptake across the cell membrane. They are the most frequent sugar transporters in bifidobacteria and have been described for mono- and oligosaccharides in different species (Nishimoto and Kitaoka, 2007; Wada et al., 2008; Wei et al., 2012), although only a few of them have been functionally characterized at protein level (Suzuki et al., 2008; Ejby et al., 2013).

Secondly, some secondary transporters, predicted to consist of single integral membrane-associated proteins, have been characterized at protein level in bifidobacteria. These include permease systems for the uptake of lactose, glucose, and sucrose (Parche et al., 2006). Secondary transporters encoding genes have been identified in different bifidobacterial species, although most of them have not been characterized at protein level (Turroni et al., 2012).

Proton symporters of the glycoside-pentoside-hexuronide (GPH) cation symporter family for melibiose and pentosides were also described in *Bifidobacterium* (Lee and O'sullivan, 2010; Turroni et al., 2012), although they remain to be characterized.

Finally, phosphoenolpyruvate-phosphotransferase (PEP-PTS) systems were first characterized in the 90's in *B. breve* and *B. bifidum* at protein level (Lee and O'sullivan, 2010). Later, genome sequence availability revealed their wide spread distribution in bifidobacteria. In particular, *B. breve* UCC2003 genome contains four PEP-PTS systems, one of which has been characterized as a fructose-specific transporter (Mazé et al., 2007). Also, *in silico* analysis revealed a putative glucose-specific PEP-PTS uptake system in *B. longum* (Lorca et al., 2007). However, genome analysis of different *B. longum* strains showed that glucose-specific PTS transporters are minor in comparison with ABC transporters (Pokusaeva et al., 2011), thus glucose may be transported preferentially by secondary permeases (Parche et al., 2006).

Comparative genomic analysis revealed that sugar PEP-PTS systems are present in all bifidobacterial genomes, except for *B. animalis* subsp. *lactis* (Lee and O'sullivan, 2010) which is hypothesized to have lost most of their carbohydrate transporters due to extended cultivation under industrial conditions. In fact, the capability to utilize variable carbon sources is considered an adaptation to the gut environment. For instance, the dominant *Bifidobacterium* species in infant fecal samples (*B. longum* and *B. bifidum*) is consistent with their inherent ability to use host-derived oligosaccharides such as mucin and HMO (Bottacini et al., 2014), and their possession of a wide range of host-derived carbohydrate transporters, such as those involved in N-biose import (Suzuki et al., 2008).

## Regulation T-effector cells and T_reg_

In the absence of disease, the ensemble of molecular interactions taking place in the human gut results in the intestinal homeostasis. Specialized epithelial cells denominated M-cells and antigen presenting cells (APCs) from the gut-associated lymphoid tissue (GALT) continuously sample the intestinal content. Interaction of APCs with the rest of GALT effectors, mainly T and B cells, leads to immunotolerance against commensal microbes and dietary components, whilst the capacity of mounting an acute, quick, and powerful response against enteropathogens is developed.

Differentiation of commensal and pathogenic bacteria is based on the presence of pattern recognition receptors (PRR) on the APC and epithelial cell surfaces. Among them, Toll-like receptors, NOD-like receptors, C-type lectin receptors, and RIG-I-like receptors are in charge of recognizing specific microbial-associated molecular patterns (MAMPs), such as flagellin, teichoic acids, or lipopolysaccharide among others. The type and intensity of the downstream and intracellular signaling cascades deployed after MAMPs-PRR interaction is essential for the APCs interaction with T-cells, which will finally determine the nature of the T-cell response. Roughly, T-cell responses are divided into effector (T_h_) and regulatory (T_reg_), its balance being key in the intestinal homeostasis maintenance (Maloy and Powrie, 2011). It is generally accepted that commensal microbiota, by inducing T_reg_ response, modulates the Th1/Th2 balance favoring immune tolerance against the gut microbiota (Ventura et al., 2012). Indeed, the classical MAMP triggering T_reg_ response is the exopolysaccharide A of the commensal bacterium *Bacteroides fragilis*, molecule also involved in the GALT maturation (Mazmanian et al., 2008).

Bifidobacteria may drive species-specific T-cell responses, as it was revealed by a series of experiments in which the cytokine secretion profiles of monocyte-derived dendritic cells (MoDCs) and full fractions of peripheral blood mononuclear cells (PBMCs) were determined (López et al., 2010). Relative levels of key cytokines (IL-10, IL-17, TNFα among others) suggested a specific immunomodulation mechanism for each species, as reported recently for probiotics (Hill et al., 2014). Challenging immature MoDCs with different strains, followed by co-culture with allogeneic naïve CD4^+^ cells and cytokine determination, further confirmed this effect (López et al., 2011).

Remarkably, *B. bifidum* LMG13195 appeared to induce a T_reg_ response *in vitro* (Figure 1; López et al., 2011). Dendritic cells challenged with membrane vesicles from this strain induced naïve CD4^+^ cells polarization into T_reg_, as deduced from the increases in the expression of *foxP3* regulation factor and the CD25 marker (López et al., 2012). Most likely, surface-associated proteins play a role in this process. Several proteins have been identified in the bifidobacterial membrane, among which moonlighting proteins such as fructose-6-phosphate phosphoketolase or enolase, might be behind the immunomodulatory effects of the membrane vesicles (Sánchez et al., 2004). However, the particular proteins involved in this T-cell polarization have not been identified.

Other immunogenic extracellular proteins are pili, proteinaceous structures that self-assemble into filaments on the bacterial surface (Ventura et al., 2012). Specifically, one sortase-dependent pili from *B. bifidum* PRL2010 induced TNFα production during transient colonization of the murine mucosa, which acted as a macrophage-activating factor during Th1 (Turroni et al., 2013). Another surface-protein able to influence T-cell responses is TgaA from *B. bifidum*, a peptidoglycan-derived enzyme able to induce DC activation and IL-2 production (Guglielmetti et al., 2014). IL-2 is one of the main cytokines supporting T_reg_ proliferation, which are characterized by the presence of CD25, the T-cell receptor for that interleukin (Zelante et al., 2012). Despite the evidence supporting an immunomodulation role of bifidobacteria, only a few surface-associated proteins have been identified as possible mediators of this effect on a limited number of *in vitro* experiments. Identifying the molecules behind this effect and confirming their efficacy in clinical trials, might provide keys to ameliorate diseases characterized by exacerbated immune responses.

## Induction of IgA production

Immunoglobulin A (IgA) is the most abundant antibody in human mucosa and modulates immune responses against commensal bacteria, preventing direct contact with immune cells (Peterson et al., 2007; Brandtzaeg, 2013). Globally, 40% of gut bacteria are IgA-coated although these values are species- and strain-dependent (Talja et al., 2014). In healthy individuals, IgA coating of bifidobacteria is higher than that of other commensals (van der Waaij et al., 2004; De Palma et al., 2010), explaining the immune tolerance to high densities of bifidobacteria. In fact, 44 proteins from *B. longum* and 24 from *B. adolescentis* were recognized by IgA (Talja et al., 2014). IgA-coated bifidobacteria also enhanced probiotic attachment to Caco-2 cells and increased production of mucosal defense molecules (Mathias et al., 2010).

Levels of IgA-coated gut commensals are altered in dysbiosis states such as those described in coeliac disease (De Palma et al., 2010), inflammatory bowel disease (van der Waaij et al., 2004), or autoimmunity disorders (Talja et al., 2014). Coeliac children showed reduced levels of bifidobacteria and IgA-coated bacteria (De Palma et al., 2010). Conversely, IgA from children developing islet autoimmunity, bound to more *B. adolescentis* antigens than those from healthy controls (Talja et al., 2014). Morevoer, bifidobacterial supplements modulate IgA production (Holscher et al., 2012; Kandasamy et al., 2014). A probiotic mixture containing bifidobacteria increased IgA and reduced diarrhea following rotavirus vaccination in a gnotobiotic pig model (Kandasamy et al., 2014). *B. animalis* Bb12 supplementation to formula-fed infants increased IgA in feces and, in those delivered by C-section, enhanced immune responses as reflected by higher anti-rotavirus and anti-poliovirus IgA production following vaccination (Holscher et al., 2012). The bifidogenic effect of galactooligosaccharides also correlated to increased IgA production (Vulevic et al., 2013; Paineau et al., 2014). Further, research to specifically delineate the bifidobacterial molecules mediating IgA induction and interaction is necessary.

## Other *Bifidobacterium* effectors of the host-microbe dialogue

A few extracellular proteins, with important physiological roles not discussed in the previous sections deserve further attention. Some *Bifidobacterium* strains produce surface-exposed Serine Protease Inhibitors of proteinaceous nature (serpins), which participate in a variety of physiological processes. The serpin produced by *B. longum* NCC2705 inhibits elastase-like proteases, including neutrophil or pancreatic elastases, thus suggesting a role in protecting bifidobacteria against exogenous proteases and potential anti-inflammatory activity (Ivanov et al., 2006). Remarkably, serpins are widely distributed in bifidobacteria and several species harbor serpin-encoding genes in their genomes (Turroni et al., 2010).

## Conclusions

Some *Bifidobacterium* proteins have been identified as mediators of the cross-talk bifidobacteria-host, providing bases to understand their beneficial traits and opening new avenues to conceive bifidobacterial-based therapeutic strategies. However, in most cases, the molecular mechanisms triggered remain unknown what has limited their translation into improved functional supplements. Identifying targets for intervention at intestinal level and developing appropriate models to search for bifidobacterial mediators is required to delineate strategies that fine-tune disease-associated alterations in the microbiota-host interplay.

## Author contributions

LR, SD, PM, AM, and BS contributed to the design and organization of the manuscript, drafted, reviewed, and accepted the final version of the manuscript.

### Conflict of interest statement

The authors declare that the research was conducted in the absence of any commercial or financial relationships that could be construed as a potential conflict of interest.
